# Supplementary material for: High throughput, label-free isolation of circulating tumor cell clusters in meshed microwells
Source: Nat Commun. 2022 Jun 13;13:3385. doi: 10.1038/s41467-022-31009-9 (PMC9192591; doi:10.1038/s41467-022-31009-9)
Supplement: Supplementary file 1 — Supplementary information [file 41467_2022_31009_MOESM1_ESM.pdf]

Supplementary Information:

High throughput, label-free isolation of circulating tumor cell clusters in meshed microwells

Mert Boya[1], Tevhide Ozkaya-Ahmadov[1], Brandi E. Swain[1], Chia-Heng Chu[1], Norh Asmare[1], Ozgun Civelekoglu[1], Ruxiu Liu[1], Dohwan Lee[1], Sherry Tobia[2], Shweta Biliya[3], L. DeEtte McDonald[3,4], Bassel Nazha[5,6], Omer Kucuk[5,6], Martin G. Sanda[7], Benedict B. Benigno[8,9], Carlos S. Moreno[5,10], Mehmet A. Bilen[5,6], John F. McDonald[3,4,8,9] and A. Fatih Sarioglu[1,3,5,8,11] *

[1]*School of Electrical and Computer Engineering, Georgia Institute of Technology, Atlanta, GA, USA*

[2]*University Gynecologic Oncology, Atlanta, GA, USA*

[3]*Parker H. Petit Institute for Bioengineering and Bioscience, Georgia Institute of Technology, Atlanta, GA, USA*

[4] *School of Biological Sciences, Georgia Institute of Technology, Atlanta, GA, USA*

[5]*Winship Cancer Institute, Emory University, Atlanta, GA, USA*

[6]*Department of Hematology and Medical Oncology, Emory University School of Medicine, Atlanta, GA, USA*

[7]*Department of Urology, Emory University School of Medicine, Atlanta, GA, USA*

[8]*Integrated Cancer Research Center, Georgia Institute of Technology, Atlanta, GA, USA*

[9]*Ovarian Cancer Institute, Atlanta, GA, USA*

[10]*Department of Pathology and Laboratory Medicine, Emory University School of Medicine, Atlanta, GA, USA*

[11]*Institute for Electronics and Nanotechnology, Georgia Institute of Technology, Atlanta, GA, USA*

*Correspondence should be addressed to A.F.S. (sarioglu@gatech.edu)

Supplementary Table 1 Number of cells observed in the input and output channels of the 2-channel microfluidic interface when it was operated in a loop without the device attached for validation.

	Input channel	Output channel
Single cells	527	528
2-cell clusters	67	66
3-cell clusters	16	15
4-cell clusters	5	5
5-cell clusters	2	2

Supplementary Table 2 Clinical characteristics of the prostate cancer patients.

* RNA-seq samples were not included in the enumeration studies as they were subjected to a different protocol including an immunostaining process that exclusively targeted cell surface markers.

Patient ID	Disease Status	Mutation profile	Local / Metastatic	PSA level (ng/mL)	Number of clusters/mL & (processed volume)	Treatment history
Patient-1 (RNA)*	Castration-resistant	Not analyzed	Metastatic	>1500	0.125 (16 mL)	ADT, taxotere, abiraterone, cabazitaxel
Patient-2 (RNA)*	Castration-resistant	Not analyzed	Metastatic	4.65	0.211 (16 mL)	ADT, enzalutamide,taxotere
Patient-3	Castration-resistant	Not analyzed	Metastatic	110.7	0.867 (15 mL)	ADT, abiraterone, enzalutamide, Radium 223, taxotere, cabazitaxel
Patient-4	Castration-resistant	Not analyzed	Metastatic	67.51	0.063 (16 mL)	ADT, abiraterone, docetaxel
Patient-5	Castration-resistant	Not analyzed	Metastatic	103.82	0.077 (13 mL)	ADT, docetaxel
Patient-6	Castration-resistant	Not analyzed	Metastatic	639	0.083 (12 mL)	ADT, enzalutamide, abiraterone, Radium 223, taxotere, cabazitaxel
Patient-7	Castration-sensitive	Not analyzed	Metastatic	1.19	0 (22 mL)	ADT
Patient-8	Castration-sensitive	Not analyzed	Metastatic	355	0 (23 mL)	ADT, abiraterone
Patient-9	Castration-resistant	Not analyzed	Metastatic	146	0.63 (17.5 mL)	ADT, abiraterone, enzalutamide, Radium 223, taxotere, cabazitaxel
Patient-10	Castration-sensitive	Not analyzed	Metastatic	5.38	0.85 (22.3 mL)	Treatment naïve

Supplementary Table 3 Clinical characteristics of the ovarian cancer patients.

* Live/Dead test sample was not included in the statistical study as it was subjected to a different protocol including an immunostaining process that exclusively targeted cell surface markers.

Patient ID	Disease Status	Mutation profile	Local / Metastatic	CA125 level (U/mL)	Number of clusters/mL & (processed volume)	Treatment history
OvC-001	High grade serous carcinoma	Not analyzed	Metastatic	3500	0.167 (6 mL)	Treatment naïve
OvC -002	High grade serous carcinoma	BRCA1, MSH6, MUTYH	Metastatic	47	1.29 (7 mL)	Had surgery, carbo/taxol/avastin; carboplatin/gemzar progressed on treatment on this date 2/15/19
OvC -003	Mixed endometrioid and clear cell carcinoma	TP53, ER/PR,MMR	Metastatic	45	3.93 (2.8 mL)	Had surgery followed by carboplatin and taxol completed 10/2018 but had progression of disease on 2/19/19
OvC -004	High grade serous carcinoma	Not identified	Metastatic	7500	0 (6.5 mL)	Had surgery carbo/taxol; avastin/carbo/doxil; Abraxane; cisplatin/gemzar progressed on treatment 2/21/19
OvC -005	Serous borderline tumor	PMS2 and PTCH1	Metastatic	13	9.73 (7.5 mL)	Treatment naïve
OvC -006	High grade serous carcinoma	NF1	Metastatic	270	4.97 (19.5 mL)	Had 3 cycles carboplatin and taxol
OvC -007	Stage IIIB High grade serous carcinoma	BRCA1	Metastatic	293	1.9 (20 mL)	Had 3 cycles carboplatin and taxol
OvC -008	Stage IIIA high grade serous carcinoma	NBN	Metastatic	1506	1.4 (20 mL)	Treatment naïve
OvC -009	Stage IIB granulosa cell tumor	Not analyzed	Metastatic	NA	59.2 (15 mL)	Had surgery followed by carboplatin and taxol in 2016, developed recurrence 6/2020
OvC -010 (Live/Dead sample)*	Stage IIIC high grade serous carcinoma	Not analyzed	Metastatic	942	0.58 (15.5 mL)	Treatment naïve

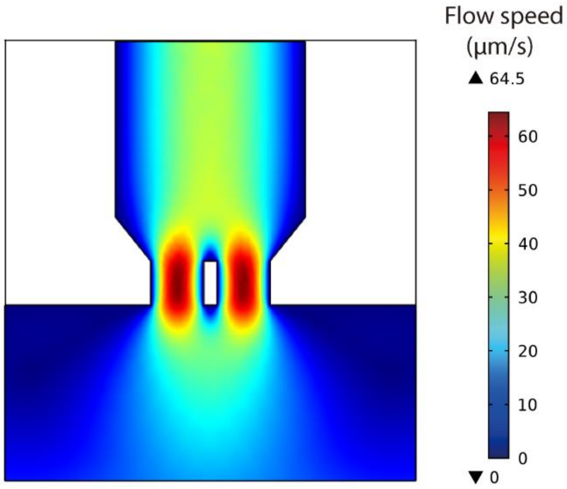

Supplementary Figure 1

Computer simulation of fluid flow within a microwell.

The plot shows the sample flow speed within an individual meshed microwell simulated with finite element analysis (COMSOL Multiphysics 5.2a). The specific simulated conditions correspond to a sample being processed with the Cluster-Wells at a volumetric flow rate of 25 mL/h. A maximum simulated flow speed of ~65 µm/s, which is still expected to be ~10X lower than physiological free flow speed in human capillaries[1], is observed at the openings of the micromesh.

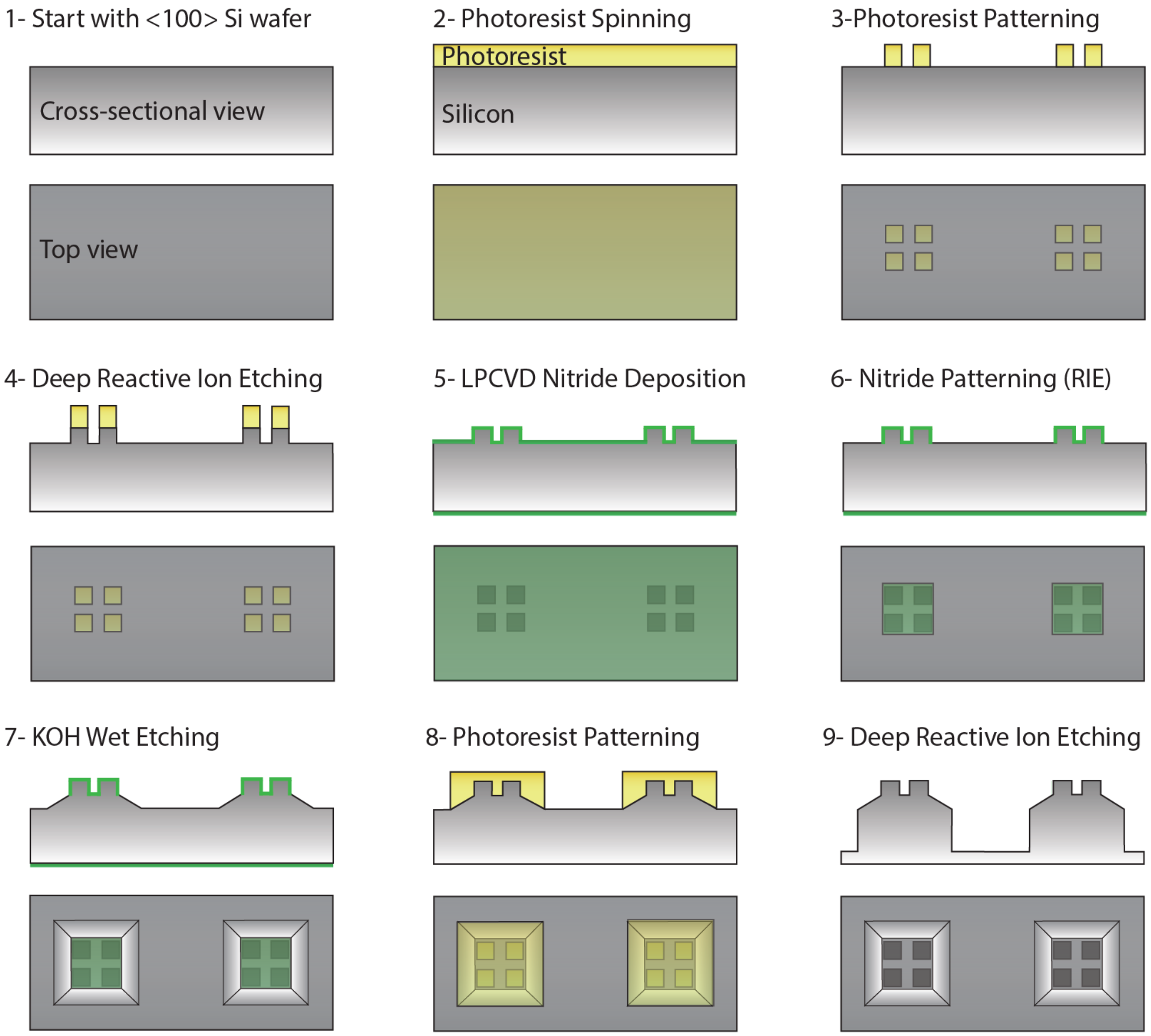

Supplementary Figure 2

Microfabrication of the silicon master-mold.

Schematic illustration of the microfabrication process used for manufacturing the silicon mold later used to create polymer-based Cluster-Wells devices. The silicon mold was patterned using a combination of photolithography, thin film deposition and dry/wet etching processes detailed by the top and cross-sectional schematics of the substrate associated with individual microfabrication steps in the figure.

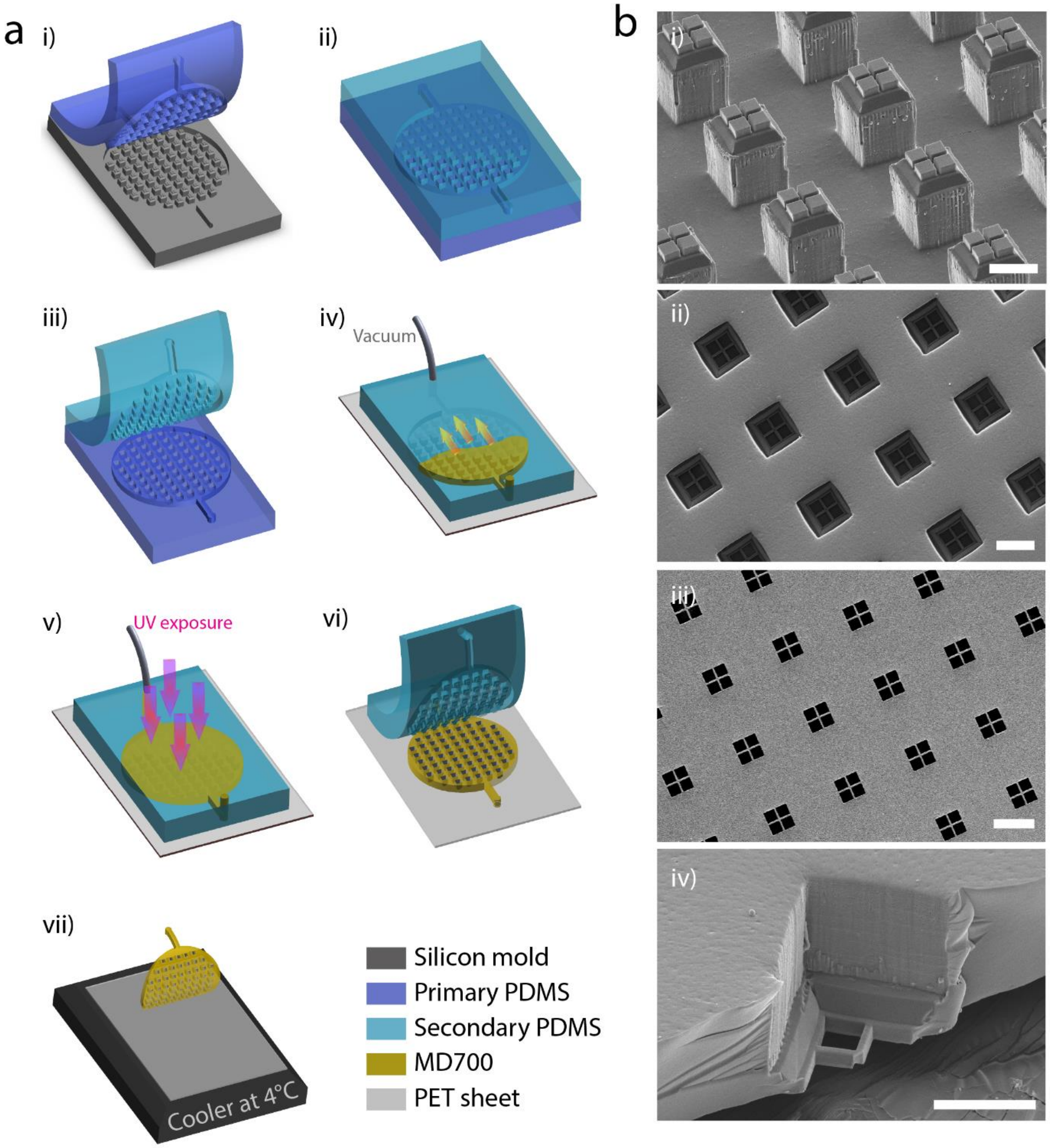

Supplementary Figure 3

Molding of the Cluster-Wells from the micromachined silicon master.

(a) Schematic illustration of the fabrication process involving soft lithography and micromolding-based techniques for the realization of polymer devices from reusable molds in a laboratory environment. The developed method eliminated the continuous need for expensive cleanroom equipment and time-consuming fabrication processes. (b) Scanning electron micrograph of (i) the PDMS mold used for patterning the Cluster-Wells (ii) the top view of fabricated polymer device (iii) the bottom view of the device (iv) the inclined cross-sectional view of an individual microwell. Scale bars, 50 μm.

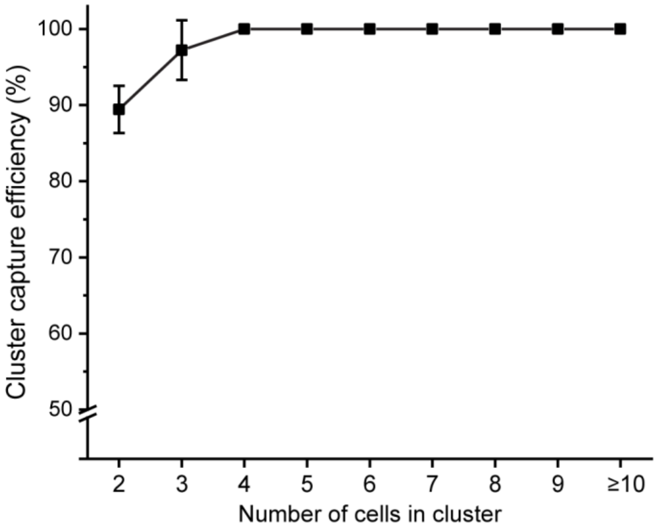

Supplementary Figure 4

Cluster-Wells capture efficiency obtained by imaging and counting the isolated clusters on the device.

Plot showing Cluster-Wells capture efficiency of spiked LNCaP prostate cell clusters processed at 25 mL/h flow rate as a function of number of cells in the cluster (n=3 independent experiments). Efficiency values from direct counting of clusters directly on the device closely matched with those values obtained with the 2-channel microfluidic interface, confirming the efficiency and reliability of the method used for characterization of the device. Data are presented as mean ± SD.

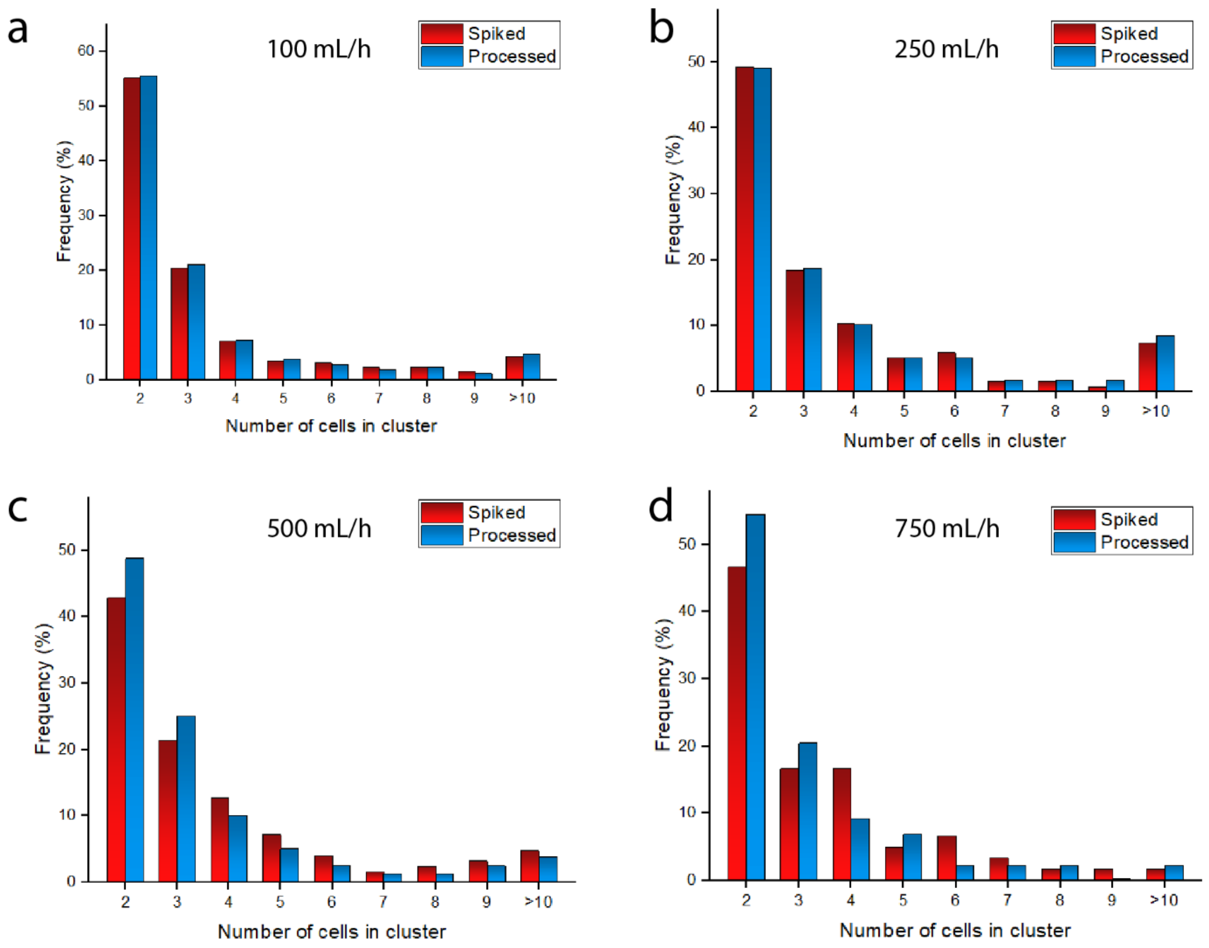

Supplementary Figure 5

Investigation of the integrity of clusters when captured at different flow rates.

Plots showing the normalized distribution of spiked and processed (captured + missed) clusters' sizes when the Cluster-Wells was operated at (a) 100 mL/h, (b) 250 mL/h, (c) 500 mL/h and (d) 750 mL/h. Matching profiles of spiked and processed cluster populations for flow rates up to 250 mL/h suggests capture of intact clusters, while the mismatch between spiked and processed populations for flow rates >500 mL/h illustrated the dissociation of larger clusters into smaller ones at those higher flow rates.

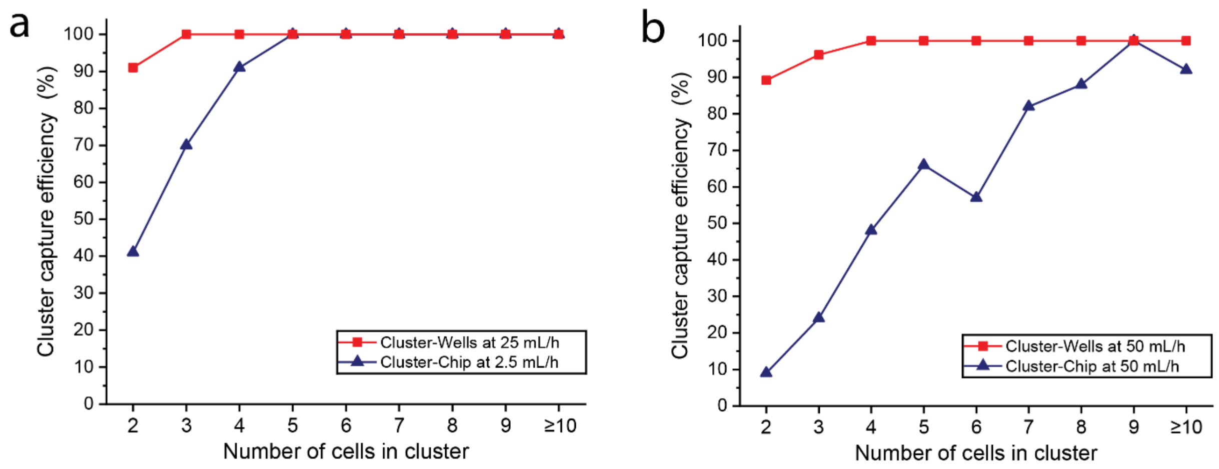

Supplementary Figure 6

Comparison of measured Cluster-Wells capture efficiencies with the published rates of the Cluster-Chip for clusters of MDA-MB-231 human breast cancer cells.

Plots showing measured cluster capture efficiencies of the Cluster-Wells together with the reported capture efficiencies of the Cluster-Chip[2] as a function of the number of cells in MDA-MB-231 clusters (a) for a matched flow speed of ~65 µm/s at cluster traps (defined as optimum conditions for both), and (b) for a matched volumetric flow rate of 50 mL/h. The capture efficiencies for Cluster-Wells experiments were performed using the experimental setup shown in Fig. 2b, while the capture efficiencies for the Cluster-Chip were taken from reference[2].

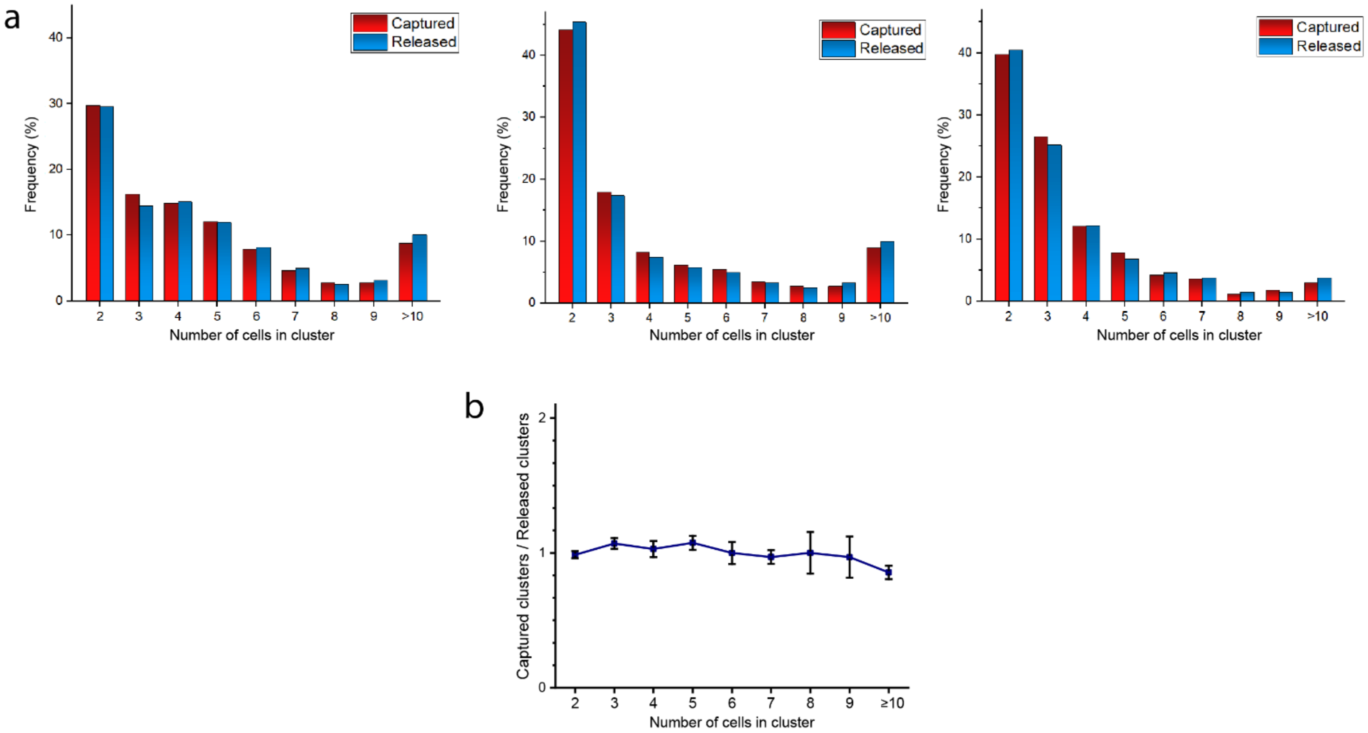

Supplementary Figure 7

Investigation of the integrity of clusters released from the Cluster-Wells.

(a) Plots showing the size distributions of captured and released clusters in three independent experiments. (b) Mean ratio of the normalized counts of captured and released clusters (n=3 independent experiments). Data are presented as mean ± SD. The match between captured and released populations illustrated that the integrity of the clusters released from Cluster-Wells was preserved.

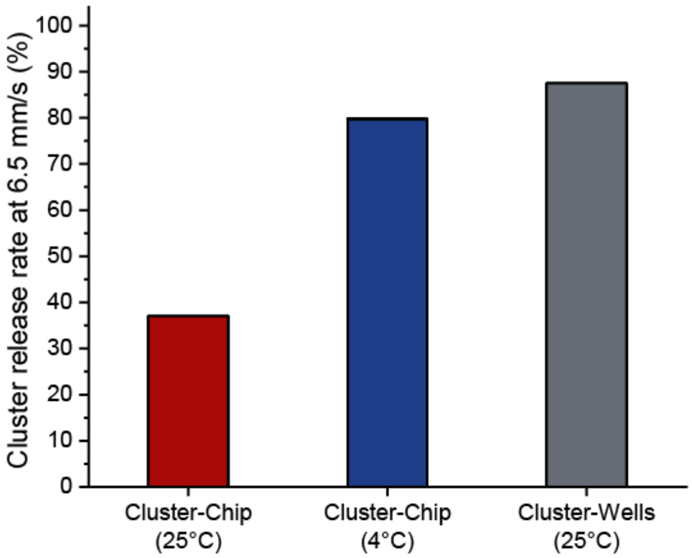

Supplementary Figure 8

Comparison of the measured Cluster-Wells release efficiencies with the published rates of the Cluster-Chip for clusters of MDA-MB-231 human breast cancer cells.

The plot shows the measured release efficiency of clusters of MDA-MB-231 cells from the Cluster-Wells together with the published release efficiency of the Cluster-Chip for MDA-MB-231 cells at 25°C and 4°C obtained from reference[2]. The Cluster-Wells release efficiency was measured under the experimental conditions that mimicked the reported conditions used for characterization of the Cluster-Chip[2], specifically by releasing MDA-MB-231 cells, initially captured at a forward flow speed of 65 μm/s, at a reverse flow speed of 6.5 mm/s.

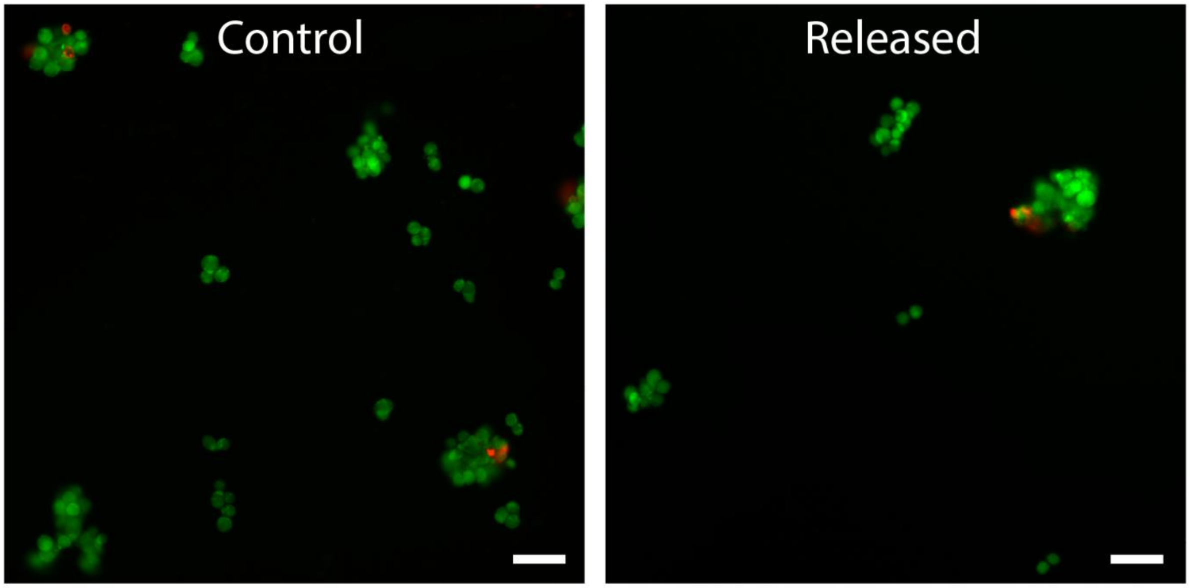

Supplementary Figure 9

Testing of the viability of LNCaP prostate cancer cell clusters enriched with the Cluster-Wells.

Fluorescence microscope images of (left) unprocessed control population of LNCaP clusters and (right) the LNCaP clusters processed using the Cluster-Wells assayed with a two color live (green) / dead (red) assay. The clusters were processed at 25 mL/h and released into a Petri dish at 6.5 mm/s reverse flow speed. The number of green (viable) and red (dead) cells were counted to compute the percentage viability of cells. Scale bars, 60 µm.

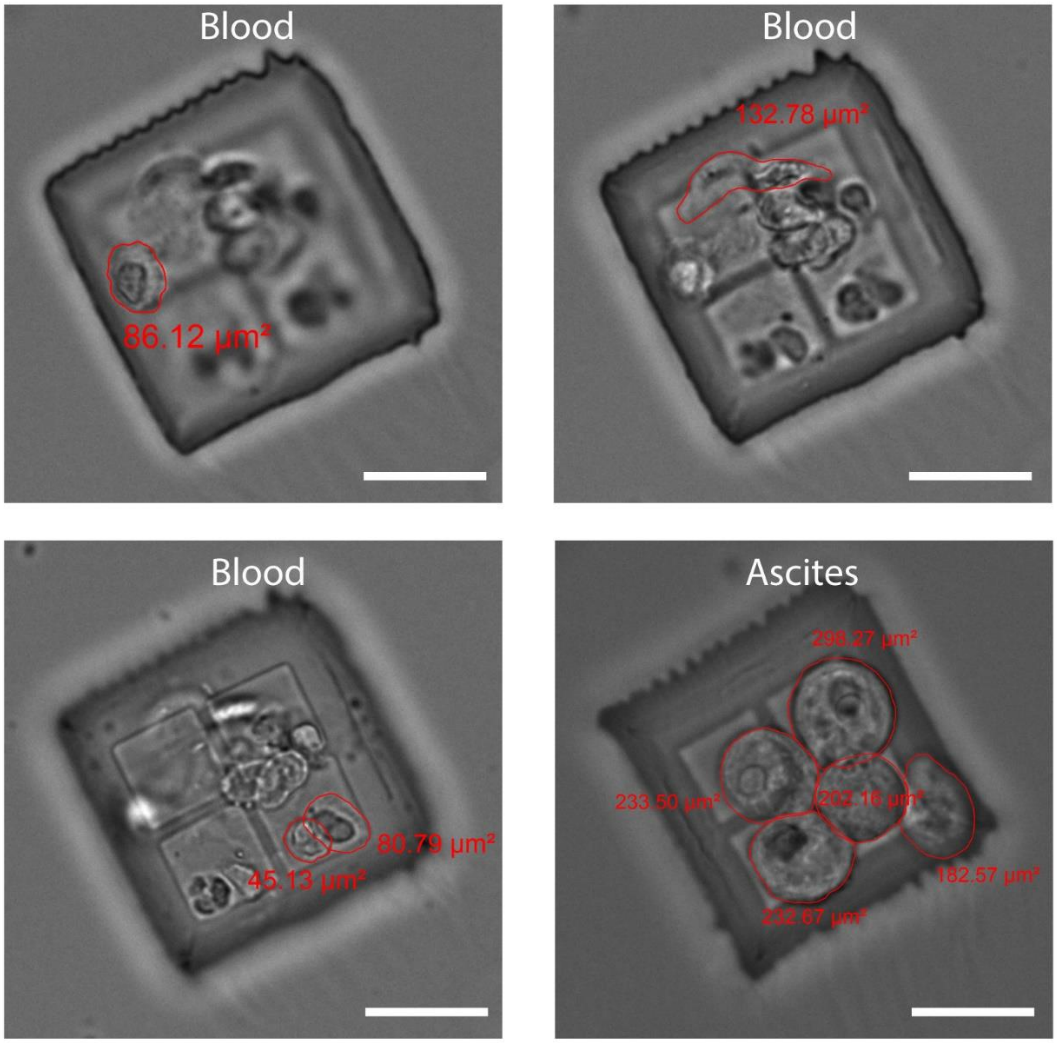

Supplementary Figure 10

Comparison of the sizes of cells in CTC clusters with those in ascites spheroids isolated from an ovarian cancer patient.

The brightfield microscope images a CTC cluster and a tumor spheroid (lower right) isolated from the same ovarian cancer patient's blood and ascites samples, respectively. Measured areas of individual cells for both were shown in the figures along with the outlined cell boundaries used for measurements. Different z-stack images of the CTC cluster were used to fully capture a cell within the same field of view. Scale bars, 20 µm.

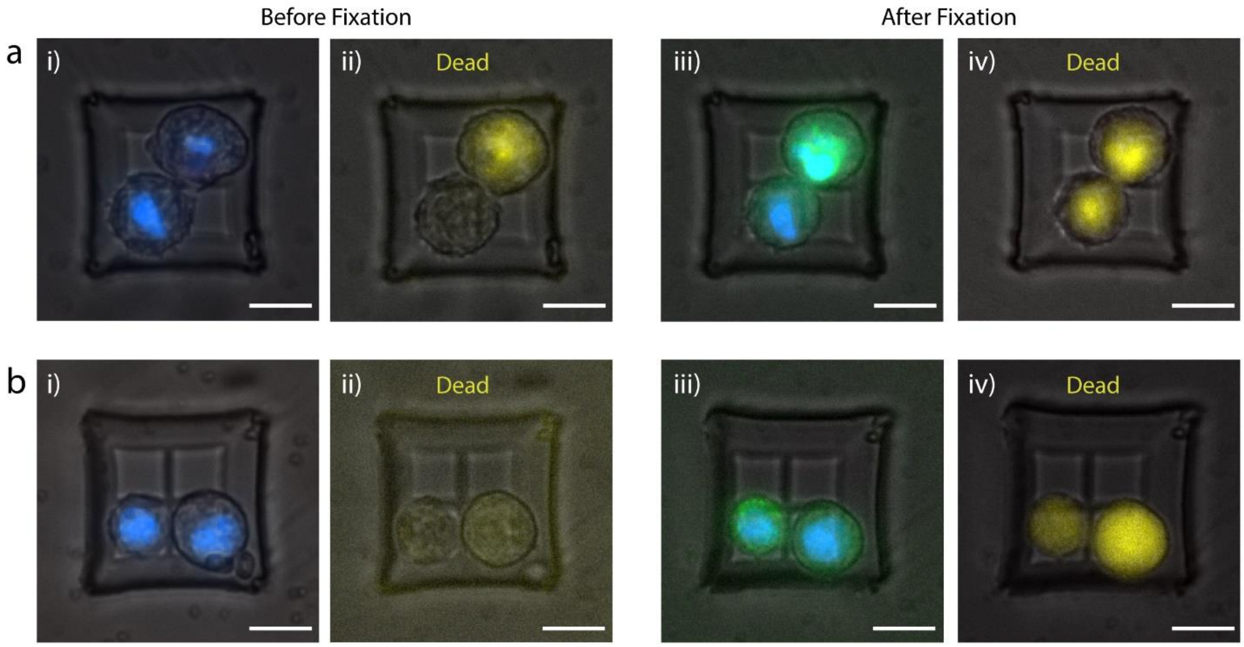

Supplementary Figure 11

Investigating the viability of CTC clusters enriched from an ovarian cancer patient.

Fluorescence images of ovarian cancer CTC clusters that are composed of (a) both live and dead CTC, and (b) only live CTCs. (i) Fluorescence images of CTC clusters whose nuclei were stained with Hoechst dye. The clusters were also subjected to PE-CD45 (TRITC) to identify any contaminating WBCs. (ii) Fluorescence images of the CTC clusters subjected to the viability assay. Dead cells were identified as the cells stained with the fluorescence assay (yellow) while viable cells were negative for the staining. (iii) Fluorescence images of the CTC clusters after they were fixed in 4% PFA and stained with antibodies against Cytokeratin 7,8/18, Vimentin and CD45. The positive staining for tumor markers (green) and negative staining for CD45 (red) proved their tumor origin. (iv) Fluorescence images of the same CTC clusters assayed with the viability after they were fixed. The positive staining and identification of fixed CTC clusters as dead validated the viability assay used in this study. Scale bars, 20 μm.

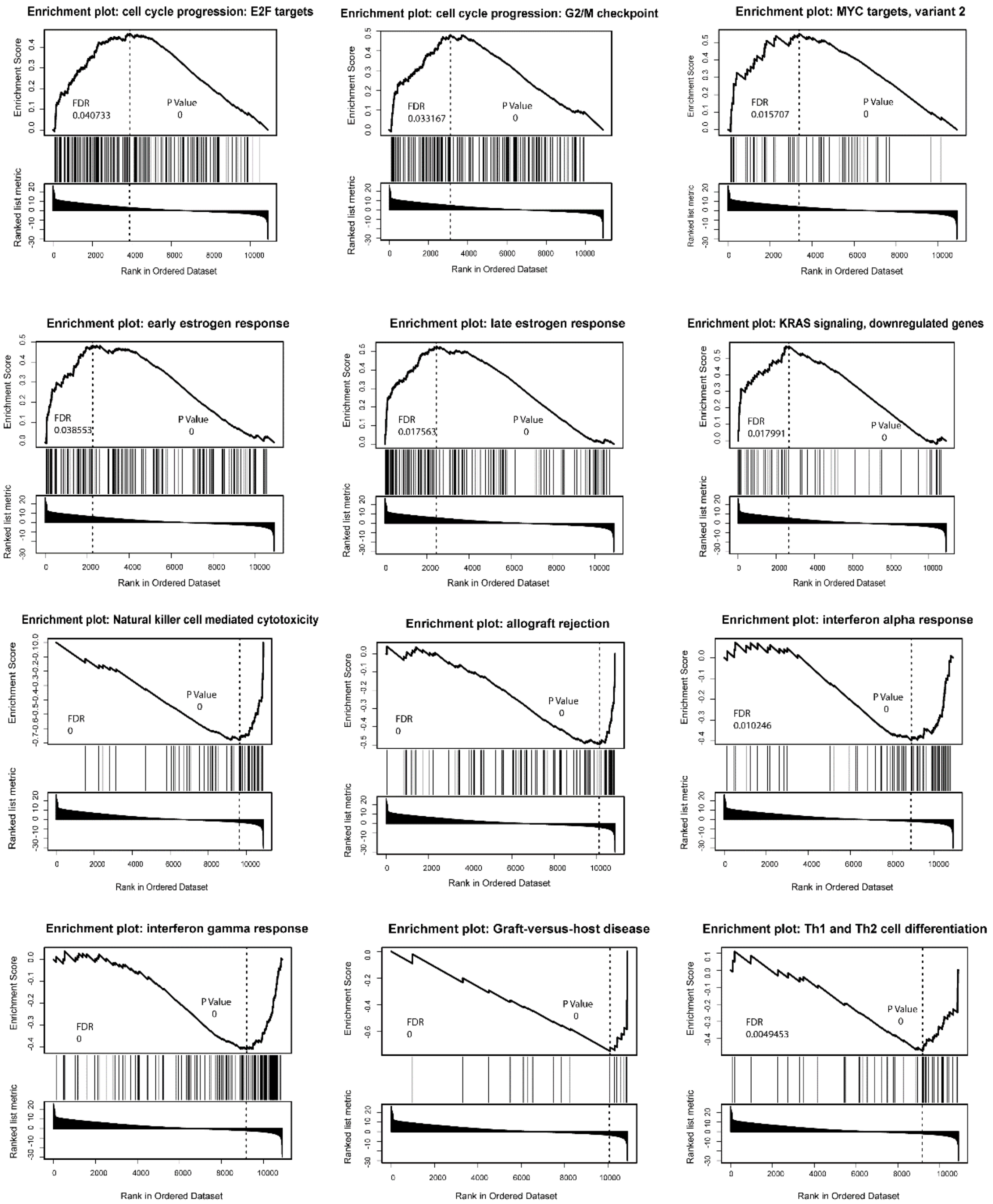

Supplementary Figure 12

Gene set enrichment analysis (GSEA) of genes differentially expressed between isolated CTC clusters and WBCs

Enrichment score plots are shown for representative significant gene sets (FDR < 0.05) from the MSigDB Hallmark 50 gene sets and the KEGG Pathway gene sets. Cancer associated gene sets (E2F targets, MYC targets, KRAS signaling, G2M checkpoint) and hormonally regulated gene sets (Estrogen early, Estrogen late) were enriched in patient clusters relative to WBC's. Immune related gene sets (allograft rejection, NK cells, IFN-gamma, and IFN-alpha responses, graft vs. host disease genes, Th1/Th2 differentiation genes) were enriched in WBC's relative to patient clusters.

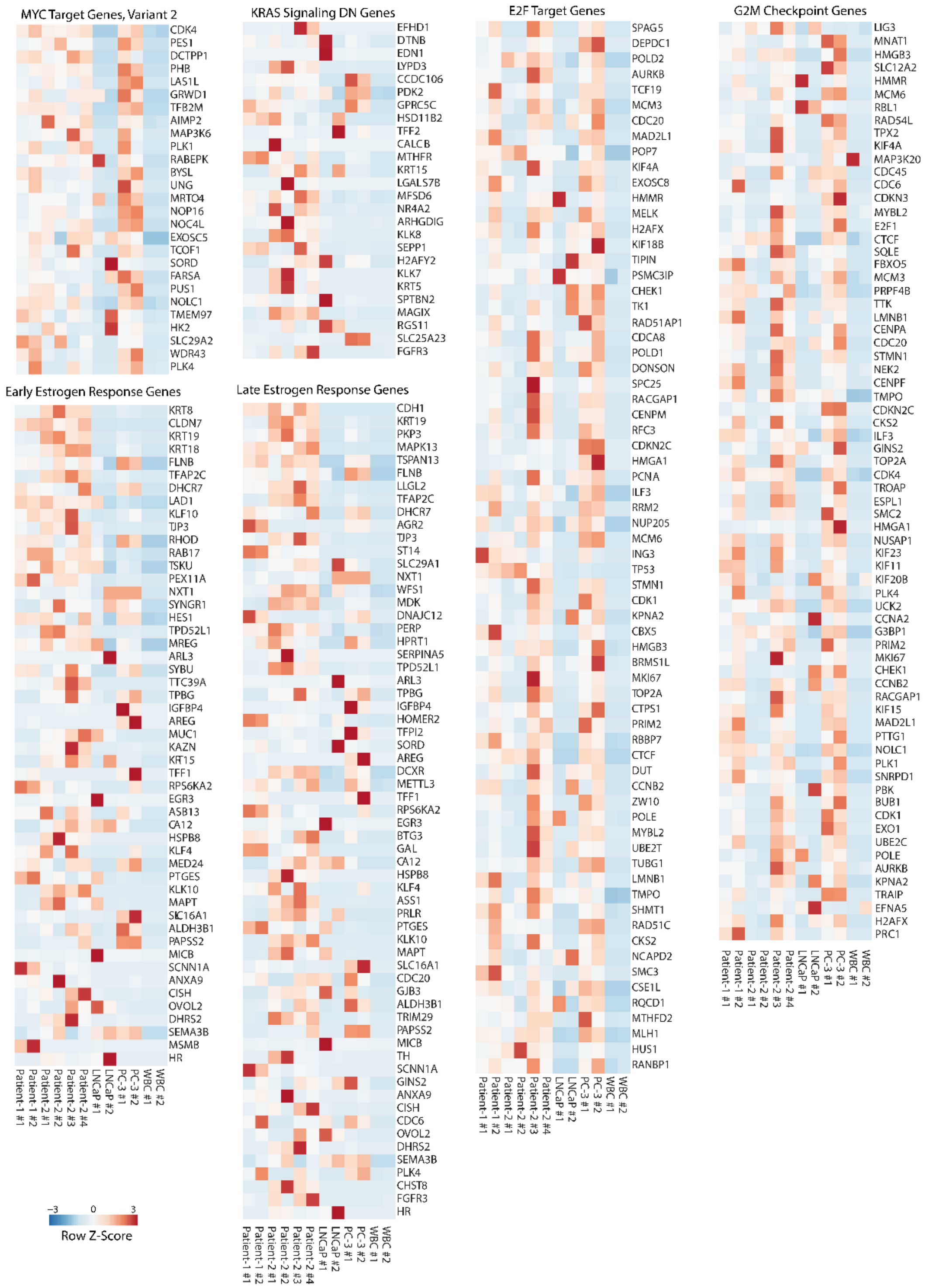

Supplementary Figure 13

Expression of genes enriched in prostate cancer CTC clusters relative to WBCs

Heatmaps showing the expression of individual genes from the five different gene sets (MYC targets, KRAS signaling, E2F targets, G2M checkpoint, Estrogen Early and Estrogen Late) significantly enriched (DESeq p-adj < 0.01) in prostate cancer CTC clusters relative to WBCs.

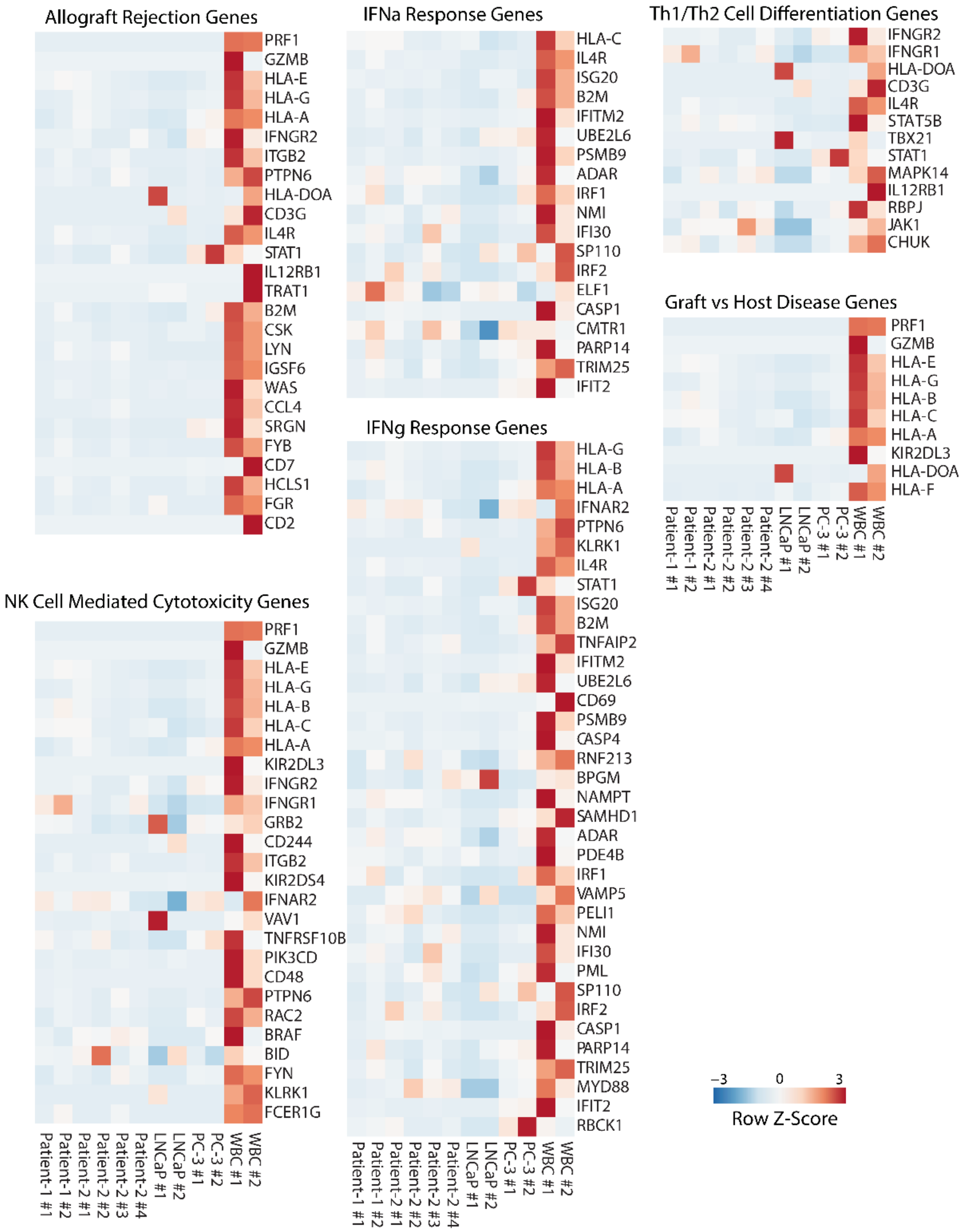

Supplementary Figure 14

Expression of genes enriched in WBCs relative to prostate cancer CTC clusters

Heatmaps showing the expression of individual genes from the six different gene sets (allograft rejection, NK cells, IFN-gamma, IFN-alpha responses, Th1/Th2 differentiation genes and graft vs. host disease genes) significantly enriched (DESeq p-adj < 0.01) in WBCs relative to prostate cancer CTC clusters.

Supplementary References

1. Stücker, M. *et al.* Capillary blood cell velocity in human skin capillaries located perpendicularly to the skin surface: Measured by a new laser Doppler anemometer. *Microvasc. Res.* **52**, 188–192 (1996).

2. Sarioglu, A. F. *et al.* A microfluidic device for label-free, physical capture of circulating tumor cell clusters. *Nat. Methods* **12**, 685–691 (2015).
