# Supplementary material for: High throughput, label-free isolation of circulating tumor cell clusters in meshed microwells
Source: Nat Commun. 2022 Jun 13;13:3385. doi: 10.1038/s41467-022-31009-9 (PMC9192591; doi:10.1038/s41467-022-31009-9)
Supplement: Supplementary file 2 — Reporting Summary [file 41467_2022_31009_MOESM2_ESM.pdf]

nature portfolio

Corresponding author(s): A. Fatih Sarioglu

Last updated by author(s): May 23, 2022

Reporting Summary

Nature Portfolio wishes to improve the reproducibility of the work that we publish. This form provides structure for consistency and transparency in reporting. For further information on Nature Portfolio policies, see our Editorial Policies and the Editorial Policy Checklist.

Statistics

For all statistical analyses, confirm that the following items are present in the figure legend, table legend, main text, or Methods section.

n/a	Confirmed	
☐	☒	The exact sample size (n) for each experimental group/condition, given as a discrete number and unit of measurement
☐	☒	A statement on whether measurements were taken from distinct samples or whether the same sample was measured repeatedly
☒	☐	The statistical test(s) used AND whether they are one- or two-sided<br>*Only common tests should be described solely by name; describe more complex techniques in the Methods section.*
☒	☐	A description of all covariates tested
☐	☒	A description of any assumptions or corrections, such as tests of normality and adjustment for multiple comparisons
☐	☒	A full description of the statistical parameters including central tendency (e.g. means) or other basic estimates (e.g. regression coefficient) AND variation (e.g. standard deviation) or associated estimates of uncertainty (e.g. confidence intervals)
☒	☐	For null hypothesis testing, the test statistic (e.g. F, t, r) with confidence intervals, effect sizes, degrees of freedom and P value noted<br>*Give P values as exact values whenever suitable.*
☒	☐	For Bayesian analysis, information on the choice of priors and Markov chain Monte Carlo settings
☒	☐	For hierarchical and complex designs, identification of the appropriate level for tests and full reporting of outcomes
☒	☐	Estimates of effect sizes (e.g. Cohen's d, Pearson's r), indicating how they were calculated

Our web collection on statistics for biologists contains articles on many of the points above.

Software and code

Policy information about availability of computer code

Data collection	Nikon NIS-Elements (4.6)
Data analysis	C++ (Visual Studio 2017), COMSOL Multiphysics (5.2a), FastQC v0.11.4 (https://www.bioinformatics.babraham.ac.uk/projects/fastqc/), WebGesalt (ref: http://www.webgestalt.org/), TrimGalore-0.6.5 (https://github.com/FelixKrueger/TrimGalore), ClustVis online tool (http://biit.cs.ut.ee/clustvis), STAR_2.5.1a (https://github.com/alexdobin/STAR), cufflinks-2.2.1.Linux_x86_64 (http://cole-trapnell-lab.github.io/cufflinks/install/), picard-tools-1.113 (https://sourceforge.net/projects/picard/files/picard-tools/), DESeq2, R version 3.6.2 (2019-12-12), WebGestalt (http://www.webgestalt.org/)

For manuscripts utilizing custom algorithms or software that are central to the research but not yet described in published literature, software must be made available to editors and reviewers. We strongly encourage code deposition in a community repository (e.g. GitHub). See the Nature Portfolio guidelines for submitting code & software for further information.

Data

Policy information about availability of data

All manuscripts must include a data availability statement. This statement should provide the following information, where applicable:

- Accession codes, unique identifiers, or web links for publicly available datasets
- A description of any restrictions on data availability
- For clinical datasets or third party data, please ensure that the statement adheres to our policy

The authors declare that all the other data supporting the findings of this study are available within the article and its supplementary information files. The raw and processed sequencing data generated in this study have been deposited in the NCBI's Gene Expression Omnibus database (GEO) under accession code GSE202650. Source data are provided as a supplementary source data file.

Publicly available datasets/tools used:
WebGestalt: http://www.webgestalt.org/
Hallmark50: https://www.gsea-msigdb.org/gsea/msigdb/genesets.jsp?collection=H
KEGG pathway: https://www.genome.jp/kegg/pathway.html
“TOMLINS_PROSTATE_CANCER_UP” gene set: https://www.gsea-msigdb.org/gsea/msigdb/cards/TOMLINS_PROSTATE_CANCER_UP
“CHANDRAN_METASTASIS_TOP50_UP” gene set: https://www.gsea-msigdb.org/gsea/msigdb/cards/CHANDRAN_METASTASIS_TOP50_UP
hg38 build of the human genome: https://www.ncbi.nlm.nih.gov/assembly/GCF_000001405.40
GRCh38.d1.vd1 Reference Sequence Hg38: https://gdc.cancer.gov/about-data/gdc-data-processing/gdc-reference-files
GenCode Annotation: gencode.v24.annotation.gtf https://www.gencodegenes.org/human/release_24.html

Field-specific reporting

Please select the one below that is the best fit for your research. If you are not sure, read the appropriate sections before making your selection.

- [x] Life sciences
- [ ] Behavioural & social sciences
- [ ] Ecological, evolutionary & environmental sciences

For a reference copy of the document with all sections, see nature.com/documents/nr-reporting-summary-flat.pdf

Life sciences study design

All studies must disclose on these points even when the disclosure is negative.

Sample size	The total number of spiked cancer cell clusters was in the range of 184 to 552 for the capture efficiency experiments. The number of spiked clusters in our experiments was set to ensure (1) the spiked cluster population was large enough to contain different-sized clusters for a comprehensive test and (2) the number of clusters did not saturate the device with the majority of the wells remaining empty. To simulate realistic conditions, cluster numbers in the range of 5 to 15 were also tested and confirmed the validity of the experimental results.
Data exclusions	No data were excluded from the study.
Replication	All characterization studies were performed with 3 independent replications and presented in the manuscript as mean ± SD.
Randomization	The fabricated devices were chosen randomly for the characterization experiments and patient sample processing. Patient samples were allocated into different groups based on their tumor origin (prostate vs ovarian).
Blinding	Blinding of investigators was not relevant for the characterization studies as the results were not subjected to individual's judgment or interpretation. Blinding was used for processing the patient blood samples. All patient information other than cancer type was withheld until the end of the study. RNA sequencing data was processed by unbiased bioinformatics pipelines prior to evaluation by the authors.

Reporting for specific materials, systems and methods

We require information from authors about some types of materials, experimental systems and methods used in many studies. Here, indicate whether each material, system or method listed is relevant to your study. If you are not sure if a list item applies to your research, read the appropriate section before selecting a response.

Materials & experimental systems

n/a	Involved in the study	
[ ]	[x]	Antibodies
[ ]	[x]	Eukaryotic cell lines
[x]	[ ]	Palaeontology and archaeology
[x]	[ ]	Animals and other organisms
[ ]	[x]	Human research participants
[x]	[ ]	Clinical data
[x]	[ ]	Dual use research of concern

Methods

n/a	Involved in the study	
[x]	[ ]	ChIP-seq
[x]	[ ]	Flow cytometry
[x]	[ ]	MRI-based neuroimaging

Antibodies

Antibodies used	Cytokeratin 8/18 Recombinant Rabbit Monoclonal Antibody from Invitrogen (Cat No: MA5-32118, Clone: SU0338, (1:400)), Purified Mouse Anti-Human CD45 from BD Biosciences (Cat No: 555480, Clone: HI30, (1:500)), PSA/KLK3 from Cell Signaling Technology (Cat No: 5365S, Clone: D6B1, (1:750)), EpCAM Recombinant Rabbit Monoclonal Antibody from Invitrogen (Cat No: MA5-29246, Clone: 28, (1:400)), PE anti-human CD45 Antibody from BioLegend (Cat No: 368510, Clone: 2D1, (1:40)), EpCAM (Alexa Fluor 488 Conjugate) from Cell Signaling Technology (Cat No: 5198S, Clone: VU1D9, (1:400)), Alexa Fluor 488 anti-human PSMA from BioLegend (Cat No: 342506, Clone: LNI-17, (1:400)), Cytokeratin 7 Recombinant Rabbit Monoclonal Antibody from Invitrogen (Cat No: MA5-32173, Clone: ST50-05, (1:400)), Vimentin Monoclonal Antibody from Invitrogen (Cat No: MA5-14564, Clone: SP20, (1:1000)), Goat anti-Rabbit IgG (H+L) Cross-Adsorbed Secondary Antibody, Alexa Fluor 488 from Invitrogen (Cat No: A-11008, Clone: NA, (1:500)), Goat

	anti-mouse IgG1 Cross-Adsorbed Secondary Antibody, Alexa Fluor 594 from Invitrogen (Cat No: A21125, Clone: NA, (1:500)), 4',6-diamidino-2-phenylindole (DAPI) (Invitrogen - Cat No: D1306, (1:1000)).
Validation	All antibodies were validated by the supplier and by references in the literature provided by the supplier for immunohistochemistry (IHC/IF), immunocytochemistry (IHC), Western Blot (WB) and flow cytometry (FC) applications using mouse and/or human samples.

Eukaryotic cell lines

Policy information about cell lines

Cell line source(s)	LNCaP, MDA-MB-231 and MCF-7 cell lines were purchased from the American Type Cell Culture (ATCC). HeyA8 cell line was provided by Dr. John F. McDonald (Georgia Institute of Technology, Atlanta, GA, USA), which was originally established and provided by Dr. Gordan Mills (MD Anderson Cancer Center, Houston, TX, USA).
Authentication	All cell lines were used within 3 months of thawing. No further authentication test was performed.
Mycoplasma contamination	Cell lines were not tested for mycoplasma contamination.
Commonly misidentified lines (See ICLAC register)	None of the cell lines used in this research are listed in the database of commonly misidentified cell lines.

Human research participants

Policy information about studies involving human research participants

Population characteristics	Characteristics of enrolled patients are shown in Supplementary Table 2 and 3. The healthy blood donors (male/female) were all older than 18 years old (age range 24 to 37).
Recruitment	The blood samples were collected from cancer patients consenting to participate in this study during their doctor visit. Metastatic patients were selected specifically for assessing the potential of the developed technology. Samples from patients were collected at Northside Hospital, Emory University Hospital and Grady Memorial Hospital. The blood collections from consenting healthy donors were performed at Georgia Institute of Technology. All samples were processed at Georgia Institute of Technology. The participants were not compensated for their enrollment.
Ethics oversight	Blood samples from consenting healthy were collected at Georgia Institute of Technology. The study included 7 healthy participants (5 male, 2 female, age range 24 to 37). The blood samples from prostate cancer patients were collected at Emory University Hospital and Grady Memorial Hospital, and the blood and ascites samples of ovarian cancer patients were collected at Northside Hospital. The study included 10 prostate cancer patients (male, age range 59 to 71) and 10 ovarian cancer patients (female, age range 27 to 78). All collections were performed under protocols approved by the Institutional Review Boards of Northside Hospital, Emory University and Georgia Institute of Technology. The participants were not compensated for their enrollment.

Note that full information on the approval of the study protocol must also be provided in the manuscript.
